# Ligation of water to magnesium chelates of biological importance

**DOI:** 10.1007/s00894-012-1459-3

**Published:** 2012-05-29

**Authors:** Dorota Rutkowska-Zbik, Małgorzata Witko, Leszek Fiedor

**Affiliations:** 1 Jerzy Haber Institute of Catalysis and Surface Chemistry, Polish Academy of Sciences, ul. Niezapominajek 8, 30-239 Krakow, Poland; 2 Faculty of Biochemistry, Biophysics and Biotechnology, Jagiellonian University, 30-387 Krakow, Poland

**Keywords:** Chlorophylls, DFT, Magnesium chelates, Porphyrins, Water

## Abstract

Water binding to several Mg^2+^ chelates, ethylenediamine, ethylenediamine-N,N’-diacetate, porphyrin, chlorophyll a and bacteriochlorophyll a, to form five- and six-coordinate complexes is studied by means of density functional theory. The results obtained for magnesium chelates are compared with the properties of the respective aqua complexes and the influence of the permittivity of environment on the ligand binding energies is discussed. Although the most common coordination number of Mg^2+^ is six, in the tetrapyrrolic chelates it is reduced to five because the accommodation of the sixth water ligand results in no gain in energy. This is in line with the experimental observations made for coordination of chlorophylls in vivo. The binding between Mg^2+^ and water is mostly of electrostatic nature, which is supported by the finding that its energy is correlated both with the electron density of the chelator and with electrostatic potential determined at the ligand binding site.

**Electronic supplementary material:**

The online version of this article (doi:10.1007/s00894-012-1459-3) contains supplementary material, which is available to authorized users.

## Introduction

Magnesium is one of the most ubiquitous metal ions in biological systems, whose role is to stabilize structures of proteins, lipid membranes, nucleotides and the nucleic acids. The stabilization is achieved by coordination of various ligands to the Mg^2+^ ion and the energy of the ligand-Mg^2+^ interactions provides the stability of the complexes. Also, Mg^2+^ is bound as the central metal ion in chlorophylls (Chls), the major photosynthetic pigments, in which it is chelated equatorially by the tetrapyrrolic macrocycle. This type of chelation does not satisfy the coordination sphere of the central Mg^2+^ ion and creates a coordination center, which can host up to two axial ligands. Thus, a coordinate bond, mainly to histidine residue, is the strongest interaction that stabilizes the structures of most photosynthetic pigment-protein complexes [1].

The coordination properties of the Mg^2+^ ion have been extensively studied both by experiment [1–5] and theory [6–13]. The most recent reviews of the theoretical investigation of the subject may be found in [14–17]. The ionic radius of the Mg^2+^ ion is relatively small (0.86 Å) and according to Pearson’s classification [18], it belongs to hard ions. As such, it forms stable complexes with O-donors, while the complexes with N-donors are somewhat less stable. In biological systems there are many types of potential ligands of Mg^2+^, such as carboxylic groups (aminoacid residues in polypeptides), carbonyl groups (from polypeptide backbone, aspargine and glutamine), amine groups (from protein backbone and lysine), imidazole moiety (from histidine), phosphate groups (in nucleotides, nucleic acids and lipids), and water molecules.

The crystallographic data base survey reveals that the preferred coordination number of Mg^2+^ is six, however the structures in which Mg^2+^ accommodates higher coordination numbers are also known [19]. The coordination number of the central Mg^2+^ ion in magnesium porphyrins and chlorophylls in free state (solution) can be either 5 or 6, depending on the ligand strength [3]. However, for Chls in vivo (bound to proteins) it rarely exceeds five [20, 21] but a six-coordinated species was also found, e.g., in the photosynthetic antenna LH1 [2]. Interestingly, to the best of our knowledge, the species with no axial ligand or with two axially ligated water molecules were never reported [1].

The reasons for the observed mismatch in Mg^2+^ behavior have already been addressed, but as yet no consistent explanation of the experimental observations was proposed. Based on the experimental results, Kania and Fiedor attribute the lack of six-coordination in Chls to a drastic change of the hardness of the central Mg^2+^ in the chelate [22]. Ryde et al. suggest that the binding of the sixth bio-ligand would not provide any gain in energy and therefore it is thermodynamically unprivileged [9]. In a recent theoretical study, Ruiz-Lopez et al. argue that the dispersion interactions play the major role in the interactions between magnesium and axial ligands in tetrapyrrolic systems and hence are the key factors controlling the axial ligation [11]. Their results are somewhat opposed to *ab-initio* HF and MP2 results on various magnesium monoligated systems as well as its hexaaqua complex, which show that by passing from HF to MP2 Mg-H_2_O interaction energy is almost unchanged [23, 24]. This discrepancy might be attributed to the fact that tetrapyrroles are more expanded, electron-rich and polarizable molecules than “simple” ligands, allowing for stronger interaction with H_2_O. Furthermore, it is known that the structure of the magnesium complexes in proteins (with acidic and neutral ligands) depends on the permittivity of the environment, what has been reviewed in details in [14]. The influence of the nature of the environment on the structural properties of the central Mg^2+^ ion in tetrapyrroles has not been addressed straightforward so far. While Ryde et al. [9] report their structures as calculated in solvent through COSMO model, Ruiz-Lopez and co-workers take no environment into consideration [11] but include one explicit water molecule interacting with H_2_O coordinated to the central magnesium ion [12].

**Fig. 1:**
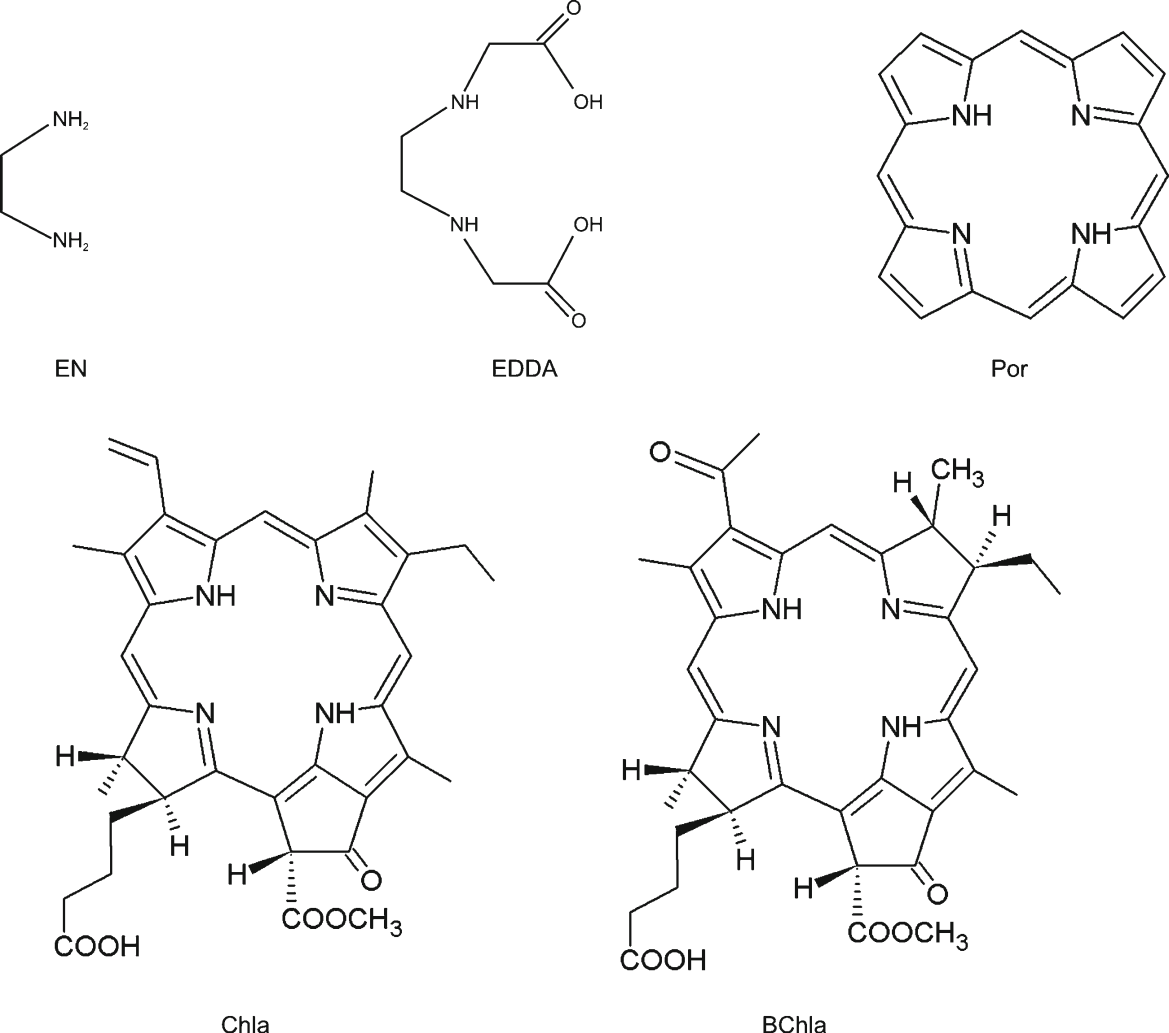
Structures of the studied ligands

In view of the above, it becomes very relevant to find out how chelation of Mg^2+^ ion by various chelating agents, not only of tetrapyrrolic type, affects its ligand binding properties. The aim of the present study is to examine in depth the effects of Mg^2+^ chelation on its interactions with an additional co-ligand, in particular a water molecule, an important biological ligand. The selected chelators contain O- and N- donors, in accordance with the chemical preferences of Mg^2+^, and include: ethylenediamine (EN), ethylenediamine-N,N’-diacetate (EDDA), porphyrin (Por), chlorophyll a (Chla) and bacteriochlorophyll a (BChla). They differ by the size, the symmetry and the degree of aromaticity (structures are shown in Fig. 1). EN is a simple bidentate N-donating ligand, which might be used to model porphyrin ligand in high accuracy quantum chemical calculations, where treating the whole porphyrin ring would be computationally too demanding. EDDA is larger, with the possibility of forming four bonds with the central magnesium anion: two with deprotonated carboxylic groups and two with nitrogen lone pairs. Por, Chla and BChla are the largest four-dentate ligands, which differ by the saturation of the pyrrolic rings in the macrocycle; two pyrroles are saturated in BChla, one in Chla, and none in Por. In the present approach, Por is void of substituents, whereas Chla and BChla posses all substituents found in their native structures with the exception of phytyl chain, substituted by a hydrogen atom. Among the studied chelates only EN is neutral while the rest of them bear −2 charge, compensating the charge of magnesium ion.

## Methods

Quantum chemical method based on density functional theory (DFT) with non-local Becke-Perdew functional [25–29] was applied to account for the interactions of the magnesium ions with selected ligands. The survey of literature data revealed that there are two most popular DFT functionals applied to study Mg^2+^ complexes: BP and B3LYP. While the latter was mainly employed to study monodentate systems, BP seems to be more often used to study tetrapyrroles, which are the important sub-group of chelates described in the manuscript. Moreover, our preliminary theoretical results showed its best performance in reproducing sixth water binding energy to [Mg(H_2_O)_5_]^2+^ (see Supporting materials). The calculation consisted of geometry optimizations of the studied structures and was further confirmed with vibrational analysis. The reported electronic energies were corrected for zero-point vibrational energy. The resolution-of-identity (RI) algorithm was applied in order to accelerate computation [30, 31]. All-electron Gaussian type orbitals of def-TZVP quality were used to define atomic orbitals [32]. The solvation was accounted for by COSMO model [33] with default radii for the elements (H = 1.30, C = 2.00, N = 1.83, O = 1.72) and 2.00 Å for magnesium. Three ε values (ε = 4, 20, 80) are used in order to take into account the nature of the possible environment in which magnesium ion is located. The low permittivity (ε = 4) represents a non-polar environment, such as the buried cavity in protein interior, higher ε value (20) relates to the cavity, which is more exposed to water, whereas the largest value represents the aqueous environment. The electronic structures of the investigated species are additionally elucidated by means of Mulliken population analysis [34]. The present results were obtained with Turbomole v. 6.3 [35].

## Results and discussion

### Reference systems: [Mg(H_2_O)_5_]^2+^ and [Mg(H_2_O)_6_]^2+^

In order to check the appropriateness of the theoretical methodology used in the present study, first the geometry and electronic structure of the five- and six-coordinate magnesium aqua complexes have been calculated – see Table 1. [Mg(H_2_O)_5_]^2+^ exhibits slightly distorted square-based pyramid geometry with all magnesium – water distances falling in the range of 2.06-2.08 Å. All Mg-water distances in octahedral [Mg(H_2_O)_6_]^2+^ are equal to 2.13 Å, c.a. 0.05 Å more than the Mg-H_2_O distances found in the crystal structures of hexaaqua magnesium species [36]. The reported binding energies (see Table 1) are calculated according to the formulae:for the fifth ligand:

$$ \Delta {\text{E}} = {{\text{E}}_{\text{tot}}}\left( {{{\left[ {{\text{Mg}}{{\left( {{{\text{H}}_2}{\text{O}}} \right)}_5}} \right]}^{{2 + }}}} \right) - {{\text{E}}_{\text{tot}}}\left( {{{\left[ {{\text{Mg}}{{\left( {{{\text{H}}_2}{\text{O}}} \right)}_4}} \right]}^{{2 + }}}} \right) - {{\text{E}}_{\text{tot}}}\left( {{{\text{H}}_2}{\text{O}}} \right), $$

and the sixth ligand:

$$ \Delta {\text{E}} = {{\text{E}}_{\text{tot}}}\left( {{{\left[ {{\text{Mg}}{{\left( {{{\text{H}}_2}{\text{O}}} \right)}_6}} \right]}^{{2 + }}}} \right) - {{\text{E}}_{\text{tot}}}\left( {{{\left[ {{\text{Mg}}{{\left( {{{\text{H}}_2}{\text{O}}} \right)}_5}} \right]}^{{2 + }}}} \right) - {{\text{E}}_{\text{tot}}}\left( {{{\text{H}}_2}{\text{O}}} \right). $$

**Table 1:** Calculated properties of [Mg(H_2_O)_5_]^2+^ and [Mg(H_2_O)_6_]^2+^. Energies are in kcal mol^-1^ and bond lengths in Å. Other published results are marked in italics: ^a^[6], ^b^[4], ^c^[23]

Complex	ΔE (gas)	ΔE (ε = 4)	ΔE (ε = 20)	ΔE (ε = 80)	r(Mg - H_2_O)
[Mg(H_2_O)_5_]^2+^	−29.4;	−18.4	−10.9	−9.7	2.06 - 2.08
	−*28.2B3LYP, -29.4MP2*;^a^				
	*exp 25.5 ± 1.3* ^b^				
[Mg(H_2_O)_6_]^2+^	−26.7	−15.6	−12.8	−11.8	2.13
	−*25.4B3LYP, -29.1MP2*; ^a^				2.10 ^c^
	*exp 23.5 ± 1.6* ^b^				

The binding energy of the fifth and sixth water in the gas phase amounts to −29.4 and −26.7 kcal mol^-1^, respectively. These values are consistently higher by 3–4 kcal mol^-1^ than the ones determined in an electrospray experiment [4].

The inclusion of the environment polarity through the COSMO model considerably lowers ligand binding energies. In water (ε = 80) it is decreased by about 60 %.

### Chelates of Mg^2+^

**Fig. 2:**
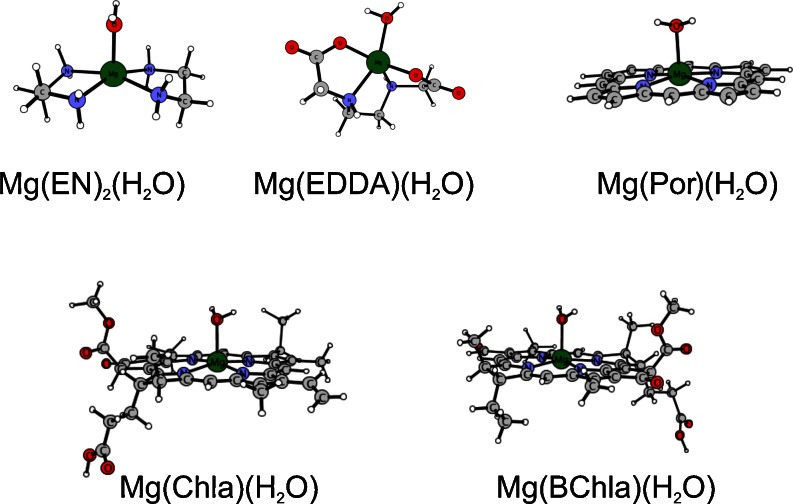
Geometry structures of the obtained five-coordinate structures

The electronic and structural parameters of the studied chelates are listed in Table 2 (five-coordinate complexes) and Table 3 (six-coordinate complexes). Geometry structures of the obtained five-coordinate complexes are shown in Fig. 2. In six-coordinate systems magnesium ion exhibits octahedral coordination. A comparison of the calculated values of the Mg-H_2_O bond lengths with the existing crystallographic and/or already published theoretical parameters is not possible for all of the investigated structures due to the lack of the appropriate data. The length of the Mg-H_2_O bond in [Mg(EDTA)(H_2_O)] is equal to 2.06 Å [19], which is shorter than the values reported in the present survey for the EDDA complexes (2.14 and 2.21 Å for the five- and six-coordinate, respectively). The computed here magnesium – water distances in all tetrapyrrolic systems are in good agreement with existing experimental and theoretical values (Tables 2 and 3). In the crystal structure of the five-coordinate [Mg(Por)(H_2_O)] complex it amounts to 2.10 Å [37]. In five-coordinate water adducts to Chla, the Mg-H_2_O bond length spans from 1.87 to 2.50 Å [38, 39], owing to the low resolution. Theoretical calculations at the DFT-BP/Ri level by Heimdal et al. [9] give 2.18 and 2.16 Å for Chla and BChla, respectively, as compared with 2.19 and 2.17 Å obtained in the present study. Ben Fredj and co-authors [11] report 2.16 and 2.24 Å for five- and six-coordinate porphyrin complexes calculated at the DFT-B3LYP level, respectively, which is slightly shorter than 2.20 and 2.26 Å reported here. As already mentioned, no crystal structures of six-coordinate water complexes of Por, Chla and BChla are known as probably no such forms exist in nature [2].

**Table 2:** Water binding energies (in kcal mol^-1^) to four-coordinate complexes of [Mg(L)_n_]^q^ type determined at BP/def-TZVP level and Mg-H_2_O bond lengths (in Å). Other published results are marked in italics: ^a^ [19], ^b^ DFT(BP/Ri) [9], ^c^ [11], ^d^ [37]

L	n	q	ΔE (gas)	ΔE (ε = 4)	ΔE (ε = 20)	ΔE (ε = 80)	Mg- H_2_O
EN	2	+2	−21.7	−13.7	−10.3	−9.6	2.13
EDDA	1	0	−17.3	−11.6	−8.8	−8.2	2.14
							*exp. 2.14* ^a^
BChla	1	0	−11.7	−9.0	−7.8	−7.5	2.17
				*−9.4* ^b^		*−8.2* ^b^	*2.16* ^b^
Chla	1	0	−11.0	−8.3	−7.1	−6.9	2.19
				*−8.5* ^b^		*−7.1* ^b^	*2.18* ^b^
Por	1	0	−10.2	−7.4	−6.3	−6.0	2.20
			*−9.5 B3LYP,*				*2.16* ^c^
			*−15.8 B3LYP + D,*				*exp. 2.10* ^d^
			*−8.9 HF,*				
			*−12.9 MP2* ^d^

**Table 3:** Water binding energies (in kcal mol^-1^) to five-coordinate complexes of [Mg(L)_n_(H_2_O)]^q^ type determined at BP/def-TZVP level and Mg-H_2_O bond lengths (in Å). Other published results are marked in italics: ^a^ [19], ^b^ [11]

L	n	q	ΔE (gas)	ΔE (ε = 4)	ΔE (ε = 20)	ΔE (ε = 80)	Mg- H_2_O
EN	2	+2	−17.6	−9.1	−5.4	−4.6	2.24
EDDA	1	0	−8.8	−3.9	−1.6	−1.0	2.21
							*exp 2.21* ^a^
BChla	1	0	−6.4	−2.3	−0.4	0.0	2.26
Chla	1	0	−6.0	−1.5	0.6	1.0	2.28; 2.29
Por	1	0	−2.7	−1.1	0.6	1.0	2.30
			*−3.8 B3LYP, -10.4 B3LYP + D,*				*2.24* ^b^
			*−1.4 HF,*				
			*−8.3 MP2* ^b^

When water molecule is coordinated by magnesium chelate to form both five- and six-coordinate adducts, the Mg-H_2_O bond is longer than the one in the reference aqua complexes. The elongation is proportional to the electron density of the ligand chelating the central ion and changes in the row: EN < EDDA < BChla < Chla < Por. Not surprisingly, the decrease of the Mg-H_2_O bond energy follows the same trend. The strongest Mg-H_2_O bond is found in the EDDA chelates, while the weakest is in Por complex. Thus, the bond strength of Mg-H_2_O in the five- and six-coordinate systems is diminished as compared to the respective aqua complexes.

Irrespective of the polarity of the environment, water binding is always thermodynamically privileged in five-coordinate complexes. In all studied chelates H_2_O binding energies are negative and in the range of typical dative water – metal bonds. As in the case of the aqua structure, Mg-H_2_O bond is weakened by 56 – 36 % (for EN and BChla, respectively) when changing from the gas phase to the aqueous solution, but still remains in the region of thermodynamically stable bonds.

The situation is somewhat different when considering six-coordinate structures. In gas phase, the electronic energy of water binding as the sixth ligand is always negative, indicating the ability to form the six-coordinate structures. The picture is changed when the environment is included in the theoretical model. The sixth ligand binding energy becomes either small, for EN and EDDA, or positive, in the case of tetrapyrrolic chelators. Interestingly, six-coordinate tetrapyrrolic adducts do not tend to be formed even in environments of very low polarity (ε = 4). This may provide an explanation for the notion that these structures are rarely found in reality - there is no net gain in energy of the whole system upon formation of six-coordinate adducts of these types.

Additionally, one should bear in mind that in aqueous media water molecules are connected by a net of hydrogen bonds. Theoretical calculations on the structure of water indicate that each H_2_O molecule forms 3.6 H-bonds on average [40–42]. The formation of another bond, such as with magnesium ion, would require breaking of at least one of these, with energetic penalty as high as 3–5 kcal mol^-1^. Also, in some cases, the bond formation might not be favored from the thermodynamic point of view, which was already discussed for magnesium [8]. As seen from experiment, an easy interchange of water molecules within its first and second coordination sphere is also observed for the simple hexaaqua magnesium complex [4]. At higher temperatures, the six-coordinate aqua complex undergoes relatively easy transformation to the four-coordinate species with two water molecules in the second coordination shell.

In view of the determined order of the decreasing binding ability of H_2_O ligand, i.e., EN < EDDA < BChla < Chla < Por, which correlates with the increasing electron density of the chelating ligand, it appears that electrostatic field of Mg chelates plays an important role in the binding of H_2_O. To assess this possibility, Mulliken and Merz-Kollman population analyses have been performed and an electrostatic potential in the same position as O atom from water ligand has been computed (water ligand was removed whereas the geometry of the rest of the system was frozen). The analysis reveals that the charge on magnesium ion and water ligands does not vary in the series of the studied complexes. The Mg charge falls in the range 1.32 - 1.36 (Mulliken) or 0.77 – 0.89 (MK), while water molecules bear net positive charge (0.06 – 0.09 according to Mulliken, and 0.02 – 0.06 according to MK). The Mg ion interacts directly with negatively charged O atoms (−0.60 − −0.51 – Mulliken, -0.86 - -0.91 - MK). The Mg-H_2_O binding energies correlate with the size of negative charge accumulated on O. The electrostatic potential at the site occupied by water molecule in the six-coordinate complex is highly positive in the EN and EDDA adducts (0.39 and 0.48 a.u., respectively). In Chla and Bchla, the electrostatic potential is lower (0.24 a.u. in each) and so are the Mg-H_2_O binding energies. For the Por complex, however, the calculated electronic potential is neutral (0.00 a.u.), not fostering the effective interaction between Mg and water. This observation is further reflected by the lowest binding energy.

All in all, it is found that for all neutral complexes water binding energies correlate with the electrostatic potentials − the correlation coefficient for the relationship is 0.99. This indicates that in this type of compounds electrostatics would prevail in Mg-H_2_O bonding, implying strong ionic character of the bonding. Moreover, the strength of the Mg-H_2_O bond is largely influenced by the interaction between H_2_O and chelator, in particular by its electronic structure. It might arise from two factors. One may be the overall electron density of the atoms forming the immediate surrounding of Mg^2+^, here, e.g., the type of the basic tetrapyrrole ring: porphyrin, chlorin (Chla), bacteriochlorin (BChla). The second is due to the presence of the substituting groups, which are further apart, but due to their polarity, may largely influence the electrostatic potential at the ligand binding site.

## Conclusions

1. Mg-H_2_O bond is longer and weaker in chelates than in the respective aqua complexes. The bond elongation and the decrease of binding energy is proportional to the electron density of the chelating ligand around the central ion and change in the following order: EN < EDDA < BChla < Chla < Por.
2. The formation of five-coordinate Mg-H_2_O complexes is thermodynamically favorable, in contrast to the formation of six-coordinate complexes, in agreement with the fact that the six-coordinate complexes of Por, Chla and BChla with water are uncommon.
3. The analysis of the factors influencing Mg-H_2_O bonding shows that the strength of Mg-H_2_O bond correlates with the negative charge of oxygen atom and the electrostatic potential at the ligand binding site. These observations lead to the conclusion that the bond between magnesium and water is mostly of electrostatic nature.
4. Water binding energies are lowered (with respect to their values in the gas phase) in all the systems when the polarity of environment is taken into account in the calculations through its dielectric constant. The higher the dielectric constant the weaker the magnesium - water bond.

## Electronic supplementary material

Below is the link to the electronic supplementary material.

ESM 1 (PDF 101 kb)
