# Supplementary material for: Ligation of water to magnesium chelates of biological importance
Source: J Mol Model. 2012 May 29;19(11):4661–7. doi: 10.1007/s00894-012-1459-3 (PMC3825143; doi:10.1007/s00894-012-1459-3)
Supplement: Supplementary file 1 — (PDF 101 kb) [file 894_2012_1459_MOESM1_ESM.pdf]

Supporting Material

Dorota Rutkowska-Zbik[1*], Małgorzata Witko[1], Leszek Fiedor[2]

Ligation of water to magnesium chelates of biological importance

[1]Jerzy Haber Institute of Catalysis and Surface Chemistry, Polish Academy of Sciences, ul. Niezapominajek 8, 30-239 Kraków, Poland

[2]Faculty of Biochemistry, Biophysics and Biotechnology, Jagiellonian University, 30-387 Kraków

*corresponding author: nczbik@cyf-kr.edu.pl, phone: +48 12 6395 155, fax: +48 12 425 19 23

Preliminary results – assessment of different DFT fuctionals for the sixth water binding energy to $[Mg(H_2O)_5]^{2+}$.

BP	B3LYP	BP+D	B97+D	Exp.
-26.7	-30.4	-29.6	-30.8	23.5 ± 1.6

Total energies corrected by ZPVE of the studied systems (in Hartrees).

System	E (gas)	E (ε=4)	E (ε=20)	E (ε=80)
$[Mg(H_2O)_4]^{2+}$	-505.412224	-505.635916	-505.723270	-505.741586
$[Mg(H_2O)_5]^{2+}$	-581.903673	-582.118312	-582.197359	-582.214643
$[Mg(H_2O)_6]^{2+}$	-658.390808	-658.591652	-658.670034	-658.686465
$[Mg(EN)_2]^{2+}$	-580.680816	-580.872074	-580.947436	-580.963303
$[Mg(EN)_2(H_2O)]^{2+}$	-657.159935	-657.346945	-657.420718	-657.436257
$[Mg(EN)_2(H_2O)_2]^{2+}$	-733.632506	-733.814483	-733.886075	-733.901136
[Mg(EDDA)]	-845.417764	-845.455067	-845.473064	-845.477147
$[Mg(EDDA)(H_2O)]$	-921.889923	-921.926543	-921.943870	-921.94777
$[Mg(EDDA)(H_2O)_2]$	-998.348567	-998.385745	-998.403139	-998.407044
[Mg(BChla)]	-2186.90403	-2186.931413	-2186.944123	-2186.946982
$[Mg(BChla)(H_2O)]$	-2263.367247	-2263.398794	-2263.413366	-2263.416633
$[Mg(BChla)(H_2O)_2]$	-2339.822008	-2339.8554	-2339.870777	-2339.87422
[Mg(Chla)]	-2149.757131	-2149.781487	-2149.792547	-2149.795008
$[Mg(Chla)(H_2O)]$	-2226.219144	-2226.247757	-2226.260725	-2226.263609

$[Mg(Chla)(H_2O)_2]$	-2302.673268	-2302.703146	-2302.716588	-2302.719571
$[Mg(Por)]$	-1188.709098	-1188.720059	-1188.724916	-1188.725984
$[Mg(Por)(H_2O)]$	-1265.169827	-1265.184891	-1265.191708	-1265.193224
$[Mg(Por)(H_2O)_2]$	-1341.618658	-1341.639696	-1341.647458	-1341.649183
H_2O	-76.444552	-76.453013	-76.456794	-76.45762588
